# Silicon microcavity arrays with open access and a finesse of half a million

**DOI:** 10.1038/s41377-019-0145-y

**Published:** 2019-04-10

**Authors:** Georg Wachter, Stefan Kuhn, Stefan Minniberger, Cameron Salter, Peter Asenbaum, James Millen, Michael Schneider, Johannes Schalko, Ulrich Schmid, André Felgner, Dorothee Hüser, Markus Arndt, Michael Trupke

**Affiliations:** 1 Faculty of Physics, University of Vienna, VCQ, Boltzmanngasse 5, 1090 Vienna, Austria; 2 Institute for Atomic and Subatomic Physics, TU Wien, VCQ, Stadionallee 2, 1020 Vienna, Austria; 3 Institute for Sensor and Actuator Systems, TU Wien, 1040 Vienna, Austria; 4 Physikalisch-Technische Bundesanstalt, Bundesallee 100, D-38116 Braunschweig, Germany; 5 Present Address: Department of Physics, King’s College London, Strand, London, WC2R 2LS UK

**Keywords:** Microresonators, Micro-optics, Quantum optics

## Abstract

Optical resonators are essential for fundamental science, applications in sensing and metrology, particle cooling, and quantum information processing. Cavities can significantly enhance interactions between light and matter. For many applications they perform this task best if the mode confinement is tight and the photon lifetime is long. Free access to the mode center is important in the design to admit atoms, molecules, nanoparticles, or solids into the light field. Here, we demonstrate how to machine microcavity arrays of extremely high quality in pristine silicon. Etched to an almost perfect parabolic shape with a surface roughness on the level of 2 Å and coated to a finesse exceeding *F* = 500,000, these new devices can have lengths below 17 µm, confining the photons to 5 µm waists in a mode volume of 88λ^3^. Extending the cavity length to 150 µm, on the order of the radius of curvature, in a symmetric mirror configuration yields a waist smaller than 7 µm, with photon lifetimes exceeding 64 ns. Parallelized cleanroom fabrication delivers an entire microcavity array in a single process. Photolithographic precision furthermore yields alignment structures that result in mechanically robust, pre-aligned, symmetric microcavity arrays, representing a light-matter interface with unprecedented performance.

Optical resonators are increasingly important tools in science and technology. Their applications range from laser physics, atomic clocks, molecular spectroscopy, and single-photon generation to the detection, trapping and cooling of atoms or nanoscale objects^1–5^. Many of these applications benefit from strong mode confinement and high optical quality factors, making small mirrors with high surface quality desirable. Building such devices with silicon yields ultra-low absorption at telecom wavelengths and enables the integration of microstructures with mechanical, electrical, and other functionalities^6,7^. Here, we push optical resonator technology^8^ to new limits by fabricating lithographically aligned silicon mirrors with ultra-smooth surfaces, small and well-controlled radii of curvature, ultra-low loss and high reflectivity. We built large arrays of microcavities with finesse values greater than *F* = 500,000 and a mode volume of 330 fL at wavelengths near 1550 nm. Such high-quality micromirrors open up a new regime of optics and enable unprecedented explorations of strong coupling between light and matter.

Scalable photonic technologies require optical devices that are compact and can be mass-produced and integrated, while maintaining high performance. Optical cavities with high field enhancement, small mode volume, and narrow linewidths are of particular importance for sideband-resolved light-matter interactions^4,9^, in transition selective photon sources^10^ or in nanoparticle cooling experiments^9,11–13^. In a two-mirror Fabry–Pérot (FP) geometry, these quantities are determined by the quality and shape of the mirror substrate, its coating and the mirror separation. Microscopic surface roughness and deviations from a perfect parabolic shape cause scattering of cavity light into higher-order modes and free space, resulting in photon loss^14^. In addition, the cavity performance depends on the mirror alignment; displacements or tilts can lead to clipping losses. For many applications, such as cooling or detection of nanoparticles, it is also important to have free access to the optical mode. This necessity is a geometric challenge in a microdesign with strict alignment requirements.

To precisely manufacture low-loss silicon mirrors with well-controlled curvature, we use a complementary metal–oxide–semiconductor compatible etching process, as depicted in Fig. 1a. The mirror geometry, including its curvature and depth, are carefully engineered by the choice of parameters in a two-step dry etching process. We achieved a surface quality approaching the atomic limit by further applying a series of oxidation and HF-etching steps^15^ (see Methods and Supplementary Information).

**Fig. 1:**
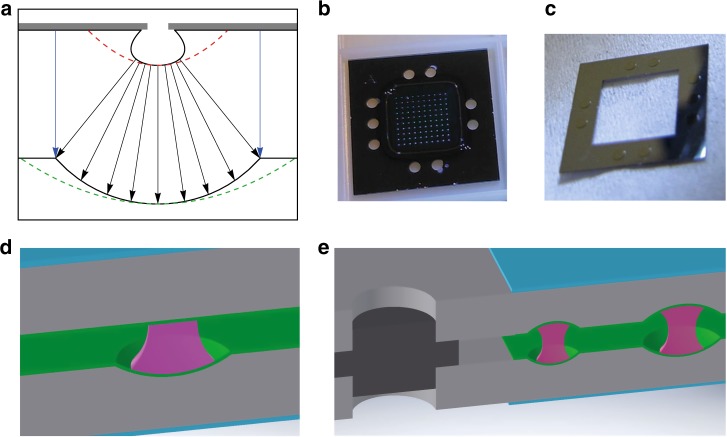
Fabrication and assembly of the micromirrors. **a** A photoresist mask (gray) is used to expose well-defined circular apertures on a silicon wafer. Plasma etching then creates concave shapes that still deviate from the desired profile (dashed red line). A second, mask-less etch step enlarges the central portion, thereby approaching an ideal parabola (green). We use a variety of aperture sizes in the initial etching mask to create a range of mirror radii of curvature. The final opening size is defined by the concurrent vertical etch (blue arrows) of the flat silicon surface. **b** A typical mirror chip (dark gray) contains 100 micromirrors of different sizes and radii of curvature, with a square pitch of 500 micrometers. It is coated with a high-reflectivity Bragg layer, which is visible as a dark-green square. Circular holes are etched into the frame to enable rigid mechanical alignment. **c** The silicon spacer with cylindrical alignment pillars matches the holes in the mirror chips. **d** Schematic of the plano-concave mirror design. A concave micromirror in silicon (gray) is combined with a plane mirror. Both are coated with the same high reflectivity multilayer Bragg stack (green). Their back sides are antireflection-coated (light blue). The two mirrors define the boundary conditions for the optical mode (purple). **e** Schematic of the symmetric silicon microcavity array with concave mirrors. The two devices are rigidly aligned and separated by an interlocking silicon spacer

The mirror chips are coated with high-reflectivity dielectric layers (*T* < 5 ppm) on their microstructured side and with an antireflection coating with a reflectivity of *ρ* < 0.1% on the other side. Figure 1b shows a chip containing 100 micromirrors, all with different curvatures to demonstrate the versatility of the method. The same coatings are applied to a set of planar silicon chips. These chips allow us to realize two different cavity geometries: plano-concave (PC) cavity arrays (see Fig. 1d), with a minimal mode volume down to 330 fL, and symmetric concave–concave (CC) cavities, with an alignment spacer (see Fig. 1e) to optimize the light-matter coupling by stronger mode confinement at the cavity center.

For both configurations, we determined the properties of the microcavity array by measuring the infrared transmission through a single-mirror pair. By scanning a laser (Toptica CTL) between 1520 nm and 1630 nm we measured the free spectral range (FSR) to determine the cavity length *L* = *c*/2*FSR*, where *c* is the speed of light (Fig. 2a). In the PC design, *L* ranges from 17 µm to 24 µm. In the CC design, *L* = 140 µm – 160 µm and can be controlled by choice of the thickness of the spacer.

**Fig. 2:**
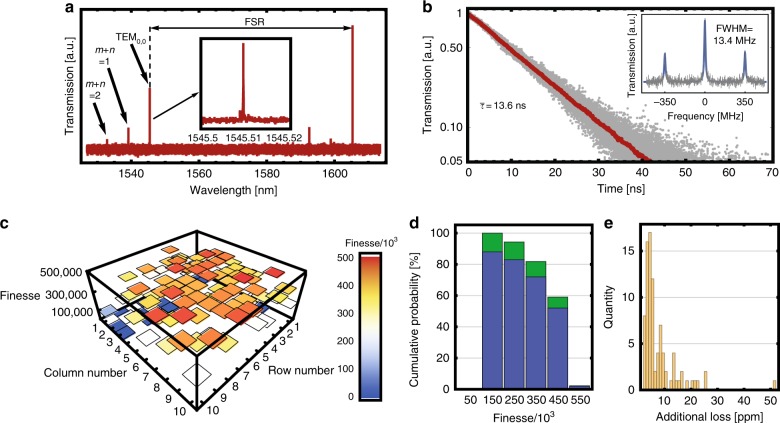
Performance of the plano-concave microcavities. **a** Spectrum of a microcavity showing the free spectral range (FSR) as well as higher-index modes which are used to determine the radius of curvature (see Methods). The inset shows the lowest-order mode. **b** A cavity ring-down measurement yields a lifetime of (13.6 ± 0.3) ns. The inset shows a single scan over the cavity resonance recorded with an applied laser frequency sideband-modulation of 350 MHz. The frequency scale given by the resulting sidebands yields a linewidth of *κ*/2*π* = (6.77 ± 0.86) MHz. With the FSR = 7.23 THz determined as in (**a**), we measure a finesse of *F* = (5.3 ± 0.6) × 10^5^ from the linewidth scan and *F* = (6.2 ± 0.2) × 10^5^ for the ring-down measurement. The uncertainties are the standard deviations over 75 and 992 measurements for the spectral and ring-down measurements, respectively. **c** Distribution of the finesse over an array of 100 microcavities. **d** Cumulative distribution of the finesse values. A resonance was found for 88 out of 100 cavities. The bars show the probability for all cavities (blue bars) and for functioning cavities (green bars). **e** Distribution of losses that we find in addition to the specified 5 ppm transmission of the mirror coatings

From the frequency separation of higher-transverse cavity modes (see Fig. 2a) we found that the mirror radii range between 123 µm and 289 µm (see Methods). To further assess the mirror quality, we measured the finesse (*F*) of the individual cavities using both cavity ring-down and sideband modulation spectroscopy. In the first scheme, a fiber-based acousto-optic modulator is used to rapidly switch off the pump laser field while the cavity is locked close to resonance. The photon lifetime or cavity decay can then be directly monitored on a fast photodiode (150 MHz bandwidth), as shown in Fig. 2b. Alternatively, we used a fiber-based electro-optic modulator to imprint well-defined frequency sidebands onto the carrier beam, which is then scanned across the cavity resonance. Using the frequency separation of the sidebands we can directly determine the cavity linewidth in a fit to the transmission spectrum, as shown in the inset of Fig. 2b.

Figure 2c shows the finesse of all 100 individual cavities on a PC array, where the etch mask radius increases from 6.2 µm to 26 µm in steps of 200 nm from row 1, column 1 to row 10, column 10. Figure 2d, e shows that a high level of performance is achieved for the entire array. Our measurements yielded a maximum finesse of *F* = (5.0 ± 0.1) × 10^5^ for *L* = 16.8 µm and *R* = 166 µm. This mirror was formed with an initial mask opening radius of 9.8 µm. This geometry results in a mode volume of only *V* = 330 fL or as little as 88*λ*^3^, while achieving an optical quality factor of *Q* = (1.1 ± 0.05) × 10^7^. These values compare favorably with those achieved for micropillar structures used to generate single photons from integrated quantum dots^16^.

Furthermore, we created rigidly assembled arrays of concave mirror pairs. The accurate and robust alignment of the cavities is achieved by applying lithographically precise micromachining to form through-etched alignment holes in the mirror chips. In a separate fabrication run, spacer chips with a thickness of 100 µm are created with 20 µm high micropillars to fit into the alignment holes. This arrangement provides precise, lithographically defined alignment and controlled spacing between the mirror chips (see Methods). The finesse for a selection of CC cavities is plotted in Fig. 3a, with a peak value of *F* = (4.0 ± 0.1) × 10^5^ for a cavity with *R* = 201 µm and *L* = 150 µm. These values correspond to *Q* = 7.9 × 10^7^. The fact that such high finesse could be achieved at a value of *L*/*R* = 0.75 indicates that the micromachined cavity pre-alignment avoids clipping losses of the optical mode at the mirror edges.

**Fig. 3:**
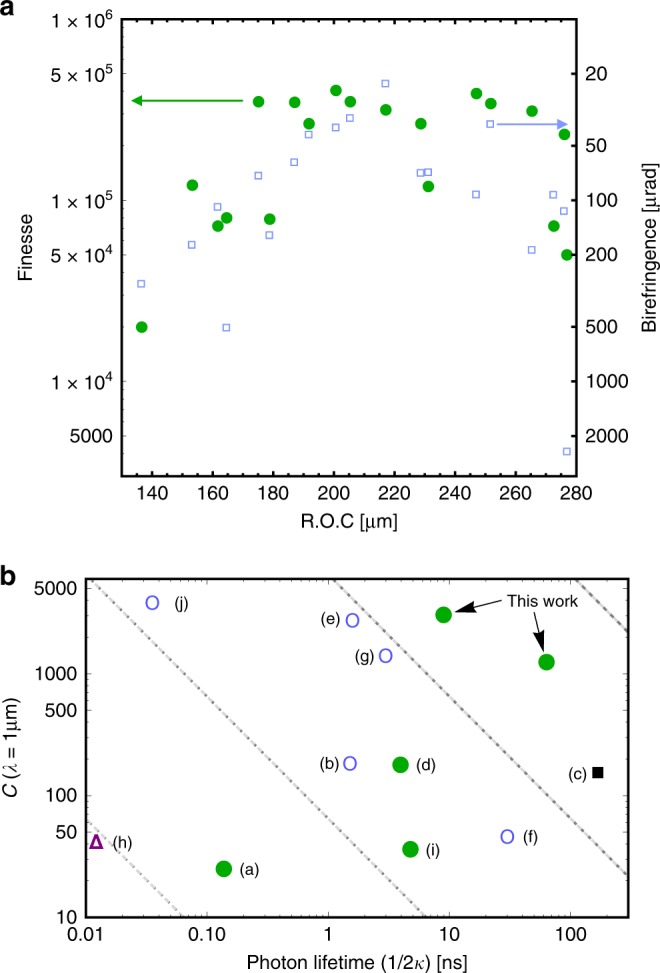
Performance of symmetric microcavities, and comparison with state of the art. **a** Finesse (green dots) and birefringence (blue squares) of all measured CC cavities. **b** Comparison of cooperativity *C* and photon lifetime for state-of-the-art microcavities ((a)^23^, (b)^24^, (c)^25^, (d)^15^, (e)^26^, (f)^17^, (g)^18^, (h)^27^, (i)^9^, and (j)^28^). Dashed lines indicate *C*(*τ*) for a constant finesse of *F* = 10^*p*^with *p* = (3–6) (left to right) and varying *L* = *R* in a symmetric cavity. Light blue circles: micromachined SiO_2_. Black square: macroscopic SiO_2_ mirrors. Purple triangle: Buckled-dome cavities. Green dots: Silicon micromirrors, including the best results from this work, as indicated, for PC (left dot) and CC (right dot) configurations

A remarkable feature of our optical resonators is their low birefringence, which is comparable to the cavity linewidth for all tested cavities. A useful measure for birefringence is the differential phase shift (*δϕ*) per roundtrip accumulated between the orthogonal polarizations of the light field. This value is given by the ratio of the frequency splitting (*δf*) between the polarization-dependent modes to the FSR: *δϕ* = 2*πδf*/FSR. It correlates with the finesse, as shown in Fig. 3a. The high performance achieved in both parameters constrains possible imperfections to a small, long range surface roughness with a spatial frequency on the order of the mode waist. For our best CC cavities, the phase shift corresponds to less than *δϕ* = 23 µrad per roundtrip. This value is comparable to the best values reported for laser machined mirror cavities^17,18^. The value suggests that *R* is nearly constant in all polar directions, which is consistent with isotropic etching and precise alignment.

A finesse of *F* > 5 × 10^5^, as found for our PC cavities, is very close to the value of *F* = 6.3 × 10^5^, which is expected from the target transmission in the coating process. Losses due to microscopic surface roughness or shape deformations must therefore be smaller than 2.8 ppm for our best cavities (see Fig. 2e and Methods). This small value must be taken as an upper limit, since it neglects residual absorption by the reflective coating and loss induced by the flat mirror. For a large fraction of CC cavities, we find a comparably low-loss value, which corroborates our assumption that the microfabrication precision fulfills its purpose and ensures high alignment quality. In the future, a finesse of *F* = 10^6^ appears possible via absorption limited coatings.

The outstanding performance of our microcavities will be of great utility for a wide range of applications. Their high finesse, strong field confinement and narrow linewidth will be important for manipulating the internal states of effective two-level systems, such as atoms or solid-state emitters or the motional states of optomechanical systems, such as levitated nanoparticles and membranes^3,4,8^.

In many applications, the relevant figure of merit is the cooperativity parameter *C* = *g*^2^/*κγ*, which compares the light-matter coupling frequency *g* in atoms, the Rabi frequency to the cavity and the matter-related damping terms *κ* and *γ*. Large values of *C* are desirable for an efficient energy exchange between the cavity and the particles. Regardless of the specific system, it can be maximized by the cavity parameters as

$$C = \frac{{3\lambda ^2}}{{\pi ^3}}\frac{F}{{w_C^2}},{\rm{ with the mode waist}}\\ w_C = \sqrt {\frac{{N_c}}{{\sqrt {\mathrm{LR}(N_c - L/R)} }}} \cdot$$

This expression is valid for PC cavities with *N*_*C*_ = 1 and for symmetric CC cavities with *N*_*C*_ = 2. Therefore, high cooperativity requires a high *F* and a strong mode confinement. For open-access, FP cavities using micromirrors, a small waist combined with a high finesse is only achievable for short cavities due to diffraction-induced clipping losses^19^. This limitation results in an inherent trade-off between photon lifetime and cooperativity.

In Fig. 3b, we plotted *C* against the photon lifetime *τ* = 1/2*κ* for a variety of microcavity systems, where we selected FP resonators with a length below 1 mm. We included micromachined and macroscopic SiO_2_ mirrors, buckled-dome cavities and silicon micromirrors. For the purpose of comparison, we recalculated all cooperativity values for *λ* = 1 µm. Due to the strong mirror curvature and high optical finesse, our cavities simultaneously attain extremely large cooperativity and photon lifetimes of several tens of nanoseconds, corresponding to MHz-range linewidths, as required for many of the applications mentioned above.

Higher cooperativity can be achieved by further reducing the mirrors’ radii-of-curvature. This reduction requires adapting the etching parameters; however, previous measurements indicate that a reduction by an order of magnitude is realistic^7^. A further increase of *C* is possible by stretching the cavity length (*L*) for longer photon lifetimes. This improvement is possible by combining a micromirror of *R* = 169 µm with a macroscopically curved substrate, e.g., with *R* = 50 mm, both coated for *F* = 5 × 10^5^. Such a device would enable a cooperativity of 2.8 × 10^3^ with a linewidth of only *κ*/*π* = 6 kHz.

In summary, the micromirrors and open-access microcavity structures presented combine extremely low losses and strong mode confinement with scalable micromachining methods and precise alignment. These features are beneficial for fundamental science and applied quantum technologies^9,20,21^. Future spacer designs can integrate quantum emitters, light guides, detector structures or optomechanical systems within the cavity frame, enabling precise overlap of the cavity field with the desired system to fully exploit the high performance of the microcavity arrays.

## Methods

### Fabrication

The micromirrors are etched into a single-crystal silicon wafer with (100) cut and weak n-doping to approximately 50 Ω cm^−1^. The etch masks are formed by adding three layers of photoresist (AZ6624), with 3 µm thickness each. The etching is performed in an SF_6_ plasma at a flow rate of 100 sccm, a temperature of 30 °C, an inductively coupled plasma power of 2 kW and a table power of 15 W. The masked etch step lasts for 320 s. The photoresist is then removed in ultrapure acetone, and the entire wafer is etched for another 45 min using the same recipe. We derive a rate of 4.2 µm/min for the masked etch. The mask-less etch rate is reduced to 0.9 µm/min due to the increased consumption of plasma by the far greater exposed silicon surface. A smoothing procedure using wet oxidation to a thickness of 2 µm, followed by oxide removal using hydrofluoric acid, is repeated twice to improve the surface quality of the mirror substrate. Bosch etching was used to create the circular holes in the chips and to separate the devices in a single step. The spacer chips and the alignment pillars on them were created by two further Bosch processes. To facilitate the final assembly, the chips were subjected to a 30 s isotropic etch. This step rounds off the edges of the pillars, and results in a reduction of the pillar radius by 0.5 µm. Taking into account this reduction, and the intrinsic precision of lithographic processing, we expect the relative positional accuracy of two opposing mirrors to be better than 1 µm.

Lastly, the microchips were secured in an aluminum mount for coating. A Bragg mirror coating, consisting of 36 alternating *λ*/4-layers of silicon dioxide (*n* = 1.45) and tantalum pentoxide (*n* = 2.04) was applied to the front of the chips. The back side was broadband antireflection coated with an optimized five-layer coating, using the same materials.

### Cavity waist

The waist of the optical mode in a FP type resonator^22^ depends on the radius of curvature of both mirrors *R*_1_ and *R*_2_ and their separation *L*

$$w_C = \sqrt {\frac{\lambda }{\pi }\sqrt {\frac{{L(R_1 - L)(R_2 - L)(R_1 + R_2 - L)}}{{(R_1 + R_2 - 2L)^2}}} } \cdot$$

This expression simplifies to the one given in the main text for a symmetric ($R_1 = R_2$) or PC cavity. The beam waist increases with distance from the focal point. On the surface of a curved mirror it is

$$w_M = w_C\sqrt {1 + \left( {\frac{{\lambda L/N_c}}{{\pi \,w_C^2}}} \right)^2} ,$$

where *N*_*C*_ = 1 for a PC geometry and *N*_*C*_ = 2 for a CC cavity geometry. The beam divergence limits the maximal possible finesse, due to the finite mirror size.

### Radius of curvature

The radius of curvature of a mirror in a FP cavity can be determined by measuring the frequency spacing of higher-order modes (see Fig. 3a). The frequency (*f*) of a mode with longitudinal index (*l*) and transverse indices (*m*, *n*) is given by

$${\hskip -14pt}f(l,m + n) = \frac{c}{{2L}}\left[ {l + \frac{{1 + m + n}}{\pi }\cos ^{ - 1}\left( {\sqrt {1 - \frac{L}{{R_1}}} \sqrt {1 - \frac{L}{{R_2}}} } \right)} \right] \cdot$$

For PC and symmetric CC cavities, the frequency difference (*χ*) between the *m* + *n* transverse mode and the fundamental mode can be divided by the free spectral range to yield

$$\chi = \, \frac{{f\left( {l,m + n} \right) - f\left( {l,0} \right)}}{{FSR}} = \frac{{m + n}}{\pi }\cos ^{ - 1}\left( {1 - \frac{L}{R}} \right)^{N_C/2} \cdot$$

The radius of curvature is then given by

$$R = \frac{L}{{1 + \left( {\cos \frac{{\pi {\mathrm{\chi }}}}{{m + n}}} \right)^{2/N_C}}} \cdot$$

### Measurement of finesse

**Fig. 4:**
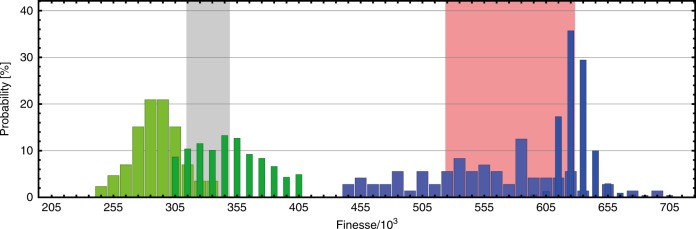
Distribution of the observed finesse values for a single, plano-concave microcavity. Finesse distributions calculated from linewidth measurements at 1604 nm (light green, broad bars) and 1545 nm (light blue, broad bars) are compared to ring-down measurements at the same wavelengths (dark green and dark blue narrow bars). The gray and light red bands show the range of finesse values between the design transmission values (9 ppm and 5 ppm) and the maximum loss due to coating imperfections (1 ppm additional scattering loss, both wavelengths)

The finesse of all cavities is determined first by measuring their linewidth. Since this method may underestimate *F* as laser noise and mechanical noise can increase the measured linewidth, we measured the photon lifetime using cavity ring-down for the selected resonators. This technique may overestimate the finesse, since electronic response functions will be convolved with the optical signal. In Fig. 4, we show an example measurement of one PC cavity. The statistics of both methods were compared to the properties of the coating, as specified by the manufacturer, taking 1 ppm of additional scattering losses into account.

The distributions confirm the expected trends for both procedures and allow us to draw two important conclusions: first, the electronic slew rate does not limit the ring-down measurements, since the significantly shorter photon lifetime at 1604 nm is clearly retrievable. Second, the finesse values stated in the main text can be considered conservative.

## Supplementary information

Supplementary Material
