# Supplementary material for: Silicon microcavity arrays with open access and a finesse of half a million
Source: Light Sci Appl. 2019 Apr 10;8:37. doi: 10.1038/s41377-019-0145-y (PMC6456601; doi:10.1038/s41377-019-0145-y)
Supplement: Supplementary file 1 — Supplementary Material [file 41377_2019_145_MOESM1_ESM.docx]

Supplementary Information for

**Silicon microcavity arrays with open access
and a finesse of half a million**

Georg Wachter (1,2), Stefan Kuhn* (1), Stefan Minniberger (1), Cameron Salter (1), Peter Asenbaum (1), James Millen (1,+), Michael Schneider (3), Johannes Schalko (3), Ulrich Schmid (3), André Felgner (4), Dorothee Hüser (4), Markus Arndt (1), and Michael Trupke*(1,2)

**Affiliations:**

1. Faculty of Physics, University of Vienna, VCQ, Boltzmanngasse 5, 1090 Vienna, Austria
2. Institute for Atomic and Subatomic Physics, TU Wien, VCQ, Stadionallee 2, 1020 Vienna, Austria
3. Institute for Sensor and Actuator Systems, TU Wien, 1040 Vienna, Austria
4. Physikalisch-Technische Bundesanstalt, Bundesallee 100, D-38116 Braunschweig

(+) Current address: Department of Physics, King's College London, Strand, London WC2R 2LS United Kingdom

**Correspondence should be addressed to:*[michael.trupke@univie.ac.at](mailto:michael.trupke@univie.ac.at) and
[stefan.kuhn@univie.ac.at](mailto:stefan.kuhn@univie.ac.at)

*Determination of losses:* The finesse of an optical cavity is limited by several factors, including the transmission *T* and absorption *A* of the mirrors, scattering losses *l_S_* due to short-scale surface roughness^1^, clipping losses *l_C_* caused by the finite aperture *R_O_* of the mirrors and mode distortion losses *l_M_* caused by large-scale mirror deformations.

The scattering and clipping losses can be estimated from

$l_{S}=1-e^{-\left( 4\pi\sigma/\lambda\right)^{2}}\cong\left( \frac{4\pi\sigma}{\lambda} \right)^{2}$ and

$$l_{c}=\frac{2}{\pi w_{M}^{2}}\int_{0}^{R_{0}} e^{-2r^{2}/w_{M}^{2}} 2\pi r dr=ⅇ^{-2R_{O}^{2}/w_{M}^{2}}.$$

The scattering losses are derived from a sinusoidal grating equation assuming white spatial noise with an rms amplitude of *σ*, while the clipping losses are calculated by integrating the power transmitted through an aperture of radius *R_O_*. We can now place bounds on the possible reductions of the reflectivity values *ρ*_1,2_ of the mirrors from the observed finesse,

$F=\frac{\pi\left( \rho_{1}\rho_{2} \right)^{1/4}}{1-\sqrt{\rho_{1}\rho_{2}}}\cong\pi\left[ T+A+\frac{l_{S,1}+l_{C,1}+l_{M,1}}{2}+\frac{l_{S,2}+l_{C,2}+l_{M,2}}{2} \right]^{-1}$.

The indices pertain to the two mirrors. We have assumed that all losses are small and that the coating properties are the same for all surfaces.

*Losses due to short-scale roughness:* Values for *l_S_* were obtained from stylus and optical profilometry, as well as atomic force microscope (AFM) measurements, which were performed after the etch procedure and after oxidation polishing.

Before polishing, profiles within a circular area of a radius of 20 µm, recorded with a tactile profilometer (Tencor P17), display a typical rms roughness of around $Rq = 2.5\pm0.8$ nm.

After smoothing by oxidation, the tactile measurement delivered an upper limit of $Rq < 2$ nm. Optical (phase-shifting interference microscope) measurements on the polished mirrors gave $Rq$ values of below $(0.5\pm0.3)$ nm. The optical measurements have an evaluated profile length of 15 µm with 60 data points. AFM measurements confirm this result: An example AFM measurement, displaying a roughness of $\sigma=0.42$ nm (rms) over a measurement area of $4\times4$ µm^2^ with $1024\times1024$ points, is shown in Fig. S1 a). The RMS roughness value $Rq$ was computed after removing a spherical shape and a line-by-line parabolic fit, but without further spectral filtering, from the standard deviation of the height values.

The properties of the substrate are expected to be perfectly replicated by the mirror coating^2^. This roughness value would therefore lead us to expect a loss of $l_{s}=10.5$ ppm, corresponding to a maximum finesse of $2.8\times{10}^{5}$. Instead, the measured finesse in both types of device points to a maximal loss of $l_{max}=2.8$ ppm, corresponding to a roughness of $\sigma_{max}=0.21$nm. It is therefore likely that not all roughness components retrieved in the AFM measurement contribute equally to the scattering loss.


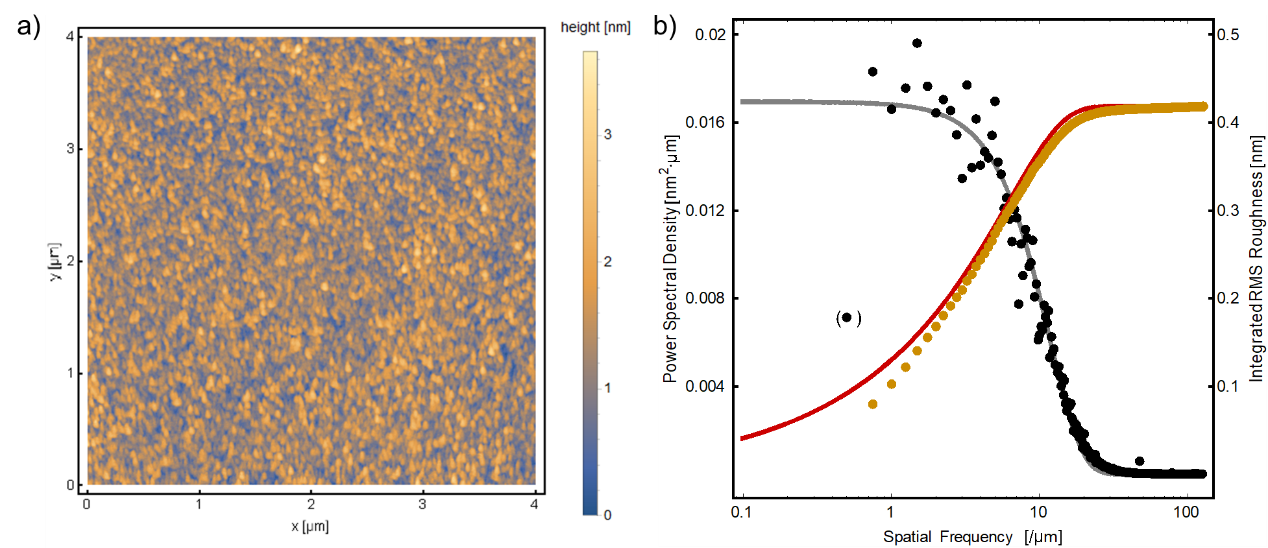


*Figure S1: Short-scale roughness of the silicon surface. a) AFM data after removal of spherical background and parabolic fit to each line. b) The extracted power spectral density of the roughness (black dots) is shown with a Gaussian fit (grey line). (The bracketed point was removed for the fit). Cumulated roughness of the spectral components (yellow dots) and corresponding function extrapolated from the fit (red line).*

The spatial power spectral density (PSD) of the amplitude noise (Fig. S1 b) of the uncoated micromirrors is closely matched by a Gaussian centred at zero,

$$PSD=P_{0}{\exp\left( -\frac{f_{s}^{2}}{f_{e}^{2}} \right)}$$

with a $1/e$-width of $f_{e}=$11.66 µm^-1^, evaluated from the average PSD of all 1024 lines. Such a spectrum indicates a Gaussian autocovariance function^3^. The step size was 4 nm per pixel and the tip radius was 10 nm, such that both enable the measurement of far higher spatial frequencies than the inverse of the observed correlation length.

Such a coloured spectrum limits the validity of the simple expression for *l_S_* used above and indicates the need for a refined analysis. Distortions on a markedly greater scale than the beam waist can be neglected: Pure sine components correspond to a tilt of the mirror, while pure cosine contributions only modify the curvature. Spatial frequencies *f_S_* smaller than the mode waist can be assumed to have the effects expected from the standard scattering approximation. However, surface amplitude noise with a spatial wavelength smaller than the optical wavelength can be expected to contribute only weakly to the loss of the specular component. We therefore cumulate the spectral components to find the maximum relevant spatial frequency of the surface roughness^4^. Assuming a hard cut-off, we calculated the resulting roughness with $\sigma_{RMS}=\sqrt{\sum_{f_{min}}^{f_{s}(max)} PSD(f_{s})/f_{min}}$, with $f_{min}=0.25$ µm^-1^, from the data and $\sigma_{RMS}=\sqrt{\frac{\sqrt{\pi}}{2}P_{0}f_{e}erf(f_{s}/f_{e})}$ by integration of the fit.

We find that the achieved finesse values can only be achieved if features with a spatial extent smaller than $f_{s}\left( \max\right)=2.65$µm^-1^, i.e. $1/f_{s}(\max)=\lambda/4.1$ do not contribute to the loss at $\lambda=1.55$ µm. Thorough analyses of the effect of roughness on the scattering properties support this reasoning, and indicate that even smaller values of $f_{s}(\max)$ should be used^3^: According to the generalized Harvey-Shack surface scatter theory, since a grating with a period smaller than $\lambda$ will not scatter light into modes with real-valued amplitude, the spectral portion $f_{s}>1/\lambda$ should be excluded. This limit yields a relevant surface roughness of $\sigma_{rel}=$0.10 nm, enabling a theoretical maximum finesse of $4.8\times{10}^{6}$.

*Losses due to shape distortions:* The mirror diameters range from 60 µm $<{2R}_{O}<$ 120 µm, making pure clipping losses negligible since $w_{M}<10$ µm for all measured cavities. Assuming a loss of $l_{max}=2.8$ ppm, the effective aperture of the mirrors is on the order of 2.53 $w_{M}$ indicating that the mirror size is not a relevant constraint. However, light scattering into higher-order modes can be related to an effective aperture of the mirrors. Since the losses due to generic mirror shape distortions cannot be calculated analytically, we determine a qualitative factor to provide insight into the characteristics of the mirrors. The ideal phase front of a Gaussian beam is a pure parabola and the lowest symmetric distortion is given by a quartic term. The additional phase shift as a function of the radial coordinate *r* is given by $\Delta\phi(r)=2Sr^{4}$. Numerical simulations^5^ indicate that a value of $S<{10}^{-7}$ is necessary in order to reach a finesse $F>5\times{10}^{5}$ in the symmetric cavity assembly. Distortions on this scale are challenging to quantify, since they require a local height resolution of nanometres on a parabolic background with a size of several micrometres. Nonetheless, the calculated limiting value of $S$ shows that the mirrors must match the desired parabolic profile to an extremely high degree.

*Birefringence:* The low birefringence observed in most of our microcavities indicates that the mirror shapes must be highly symmetric. The observed frequency splitting of the orthogonal polarization states could be related to mirror imperfections caused by the etching process, but also due to mirror misalignment or tilt. It was previously shown^6^ that a difference in radii of curvature (along two orthogonal axes) can lead to such a splitting, where the length-independent phase shift is given by

$\delta\phi=\frac{1}{k}\frac{1-R_{X}/R_{Y}}{R_{X}}$.

For a symmetric CC cavity with a radius of curvature along one azimuthal axis $R_{X}=201$ µm, and assuming equal shifts on both surfaces, this expression gives an orthogonal radius $R_{Y}=202.9$ µm. The observation of birefringence does not necessarily require an asymmetry of the shape itself, but can also be caused by an off-axis displacement of the cavity mode spot: The local radius of curvature of a parabola with $z=(x^{2}+y^{2})/2R(0)$ is given by $R_{Y}(y)=R(0)\times\left[ 1+\frac{y^{2}}{{R(0)}^{2}} \right]^{3/2}$. The orthogonal radius of curvature increases more slowly, with $R_{X}(y)=R(0)\times\left[ 1+\frac{y^{2}}{{R(0)}^{2}} \right]^{1/2}$. For perfectly parabolic mirrors, an off-axis displacement of their centres of 19.5 µm, corresponding to a tilt of the cavity axis by 15°, would be required to cause such a splitting. This displacement or tilt is more than an order of magnitude greater than expected from our fabrication tolerances and is therefore not expected to be the dominant contribution to the observed splitting. Aside from assembly effects, strain in the coating can contribute to the birefringence at the level of several µrad^7^.

Finally, the strong correlation between finesse and birefringence (see main text, Fig. 3a) indicates a common cause: It is likely that both arise from residual shape deviations with a spatial wavelength on the order of the mode size, since such distortions can lead to scattering into higher-order modes and to a small angular dependence of the radius of curvature^5,6^.

1. Beckmann, P. & Spizzichino, A. *The scattering of electromagnetic waves from rough surface*. (Pergamon Press, 1963).

2. Amra, C. Light scattering from multilayer optics II Application to experiment. *J. Opt. Soc. Am. A* **11,** 211 (1994).

3. Harvey, J. E., Choi, N., Schroeder, S. & Duparré, A. Total integrated scatter from surfaces with arbitrary roughness, correlation widths, and incident angles. *Opt. Eng.* **51,** 013402 (2012).

4. Lawson, J. K. *et al.* Specification of optical components using the power spectral density function. in (eds. Doherty, V. J. & Stahl, H. P.) 38–50 (1995). doi:10.1117/12.218430

5. Kleckner, D., Irvine, W. T., Oemrawsingh, S. S. & Bouwmeester, D. Diffraction-limited high-finesse optical cavities. *Phys. Rev. A* **81,** 043814 (2010).

6. Uphoff, M., Brekenfeld, M., Rempe, G. & Ritter, S. Frequency splitting of polarization eigenmodes in microscopic Fabry–Perot cavities. *New J. Phys.* **17,** 013053 (2015).

7. Asenbaum, P. & Arndt, M. Cavity stabilization using the weak intrinsic birefringence of dielectric mirrors. *Opt. Lett.* **36,** 3720 (2011).
